# Practical Indications and Intraoperative Contribution of Neuroendoscopic Assistance During Exoscopic Skull Base Surgery: A Single-Center Experience

**DOI:** 10.3390/brainsci16090942

**Published:** 2026-09-03

**Authors:** Fumihiro Matano, Miku Tsuruya, Satsuki Imaoka, Kaoru Namatame, Megumi Tomita, Yohei Nounaka, Takao Kitamura, Kazutaka Shirokane, Katsuya Umeoka, Yasuo Murai

**Affiliations:** Department of Neurological Surgery, Nippon Medical School, 1-1-5 Sendagi, Bunkyo-ku, Tokyo 113-8603, Japan; s16-069tm@nms.ac.jp (M.T.); 17satsuki.i.01@nms.ac.jp (S.I.); t-megumi@nms.ac.jp (M.T.); y-nonaka@nms.ac.jp (Y.N.); taka-kitamu@nms.ac.jp (T.K.); ymurai@nms.ac.jp (Y.M.)

**Keywords:** neuroendoscopy, exoscope, skull base surgery, aneurysm surgery, endoscope-assisted surgery, surgical ergonomics, microsurgery

## Abstract

Background: Neuroendoscopy complements exoscopic visualization by providing angled views of anatomical structures hidden from the direct surgical line of sight. However, its practical indications and intraoperative contribution during exoscope-assisted skull base surgery have not been systematically evaluated. This study aimed to characterize the patterns of neuroendoscopic use and its observed intraoperative contribution during exoscope-assisted skull base surgery. Methods: We retrospectively reviewed 164 consecutive skull base procedures performed between March 2024 and June 2026. After excluding 58 pure endoscopic endonasal procedures, 106 exoscope-assisted procedures were analyzed. Neuroendoscopic assistance was used in 42 procedures, each performed in a different patient. Complete operative video recordings from these 42 procedures were retrospectively reviewed by two neurosurgeons, and the observed contribution of neuroendoscopy was classified as visualization-only, confirmatory, or decision-changing. Results: Neuroendoscopic assistance was used in 42 of 106 exoscope-assisted procedures (39.6%), including 30 aneurysms and 12 tumors. The most common indication was clip and perforator confirmation during aneurysm surgery (30/42, 71.4%). The observed contribution was classified as visualization-only in 8 procedures (19.0%), confirmatory in 20 (47.6%), and decision-changing in 14 (33.3%). Among the 14 decision-changing procedures, neuroendoscopic findings led to clip repositioning in 5 aneurysm procedures and additional tumor removal in 9 tumor procedures. No complications judged attributable to neuroendoscope insertion or manipulation were identified in the 42 procedures. Conclusions: Neuroendoscopic assistance provided additional intraoperative information during exoscope-assisted skull base surgery, with decision-changing findings identified in 14 of 42 assisted procedures. These findings characterize the observed contribution and practical indications of neuroendoscopic assistance; however, the retrospective design, selective use of neuroendoscopy, and limited sample size preclude conclusions regarding comparative efficacy or safety.

## 1. Introduction

Neuroendoscopy has expanded the surgical armamentarium for skull base lesions by providing angled visualization of anatomical structures hidden from the direct line of sight [1,2]. In particular, neuroendoscopy is useful for the inspection of deep neurovascular structures, including the optic canal, Meckel’s cave, and perforating arteries, and has been widely adopted as an adjunct to microscopic surgery [3].

More recently, exoscopes have gained popularity in neurosurgery because of their excellent image quality, improved ergonomics, and wider working space [4]. Compared with conventional operating microscopes, exoscopes provide a longer focal distance and a smaller optical head, facilitating the introduction and manipulation of neuroendoscopes within the surgical corridor [5]. Furthermore, simultaneous display of exoscopic and endoscopic images, including picture-in-picture and side-by-side display modes, allows concurrent visualization of both image sources [6]. These characteristics may further enhance the utility of neuroendoscopy during skull base surgery. Although several preliminary reports have described the simultaneous use of exoscopes and neuroendoscopes, the clinical role and practical indications of neuroendoscopic assistance during exoscope-assisted skull base surgery have not been systematically investigated [7].

Therefore, the present study aimed to characterize the patterns of neuroendoscopic use, practical indications, and observed intraoperative contribution of neuroendoscopic assistance during exoscope-assisted skull base surgery. We also describe the operative setup for combined exoscopic and neuroendoscopic visualization and discuss practical situations in which neuroendoscopic assistance may be considered.

## 2. Methods

### 2.1. Study Design and Patient Selection

**Figure 1:**
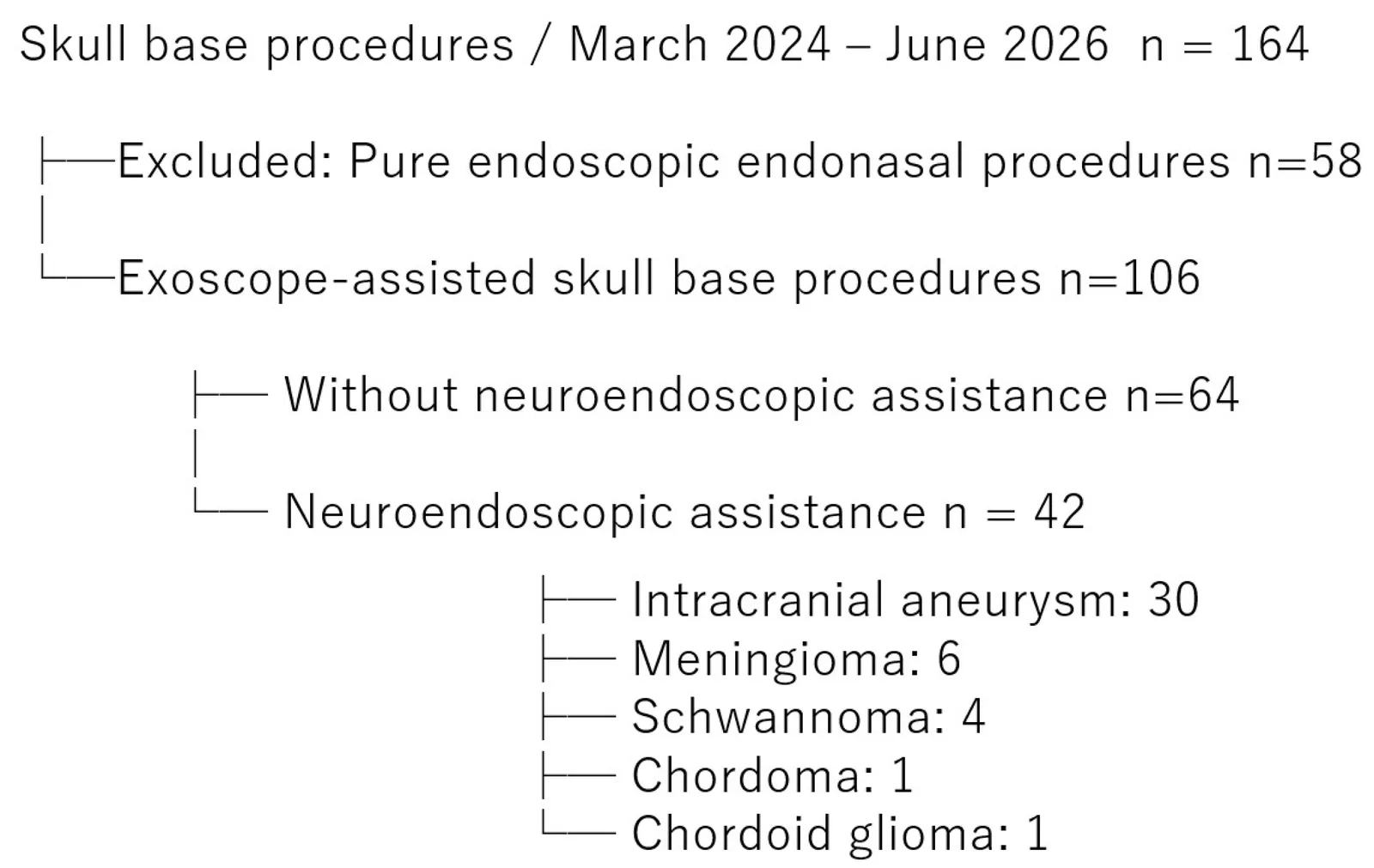
Study flow diagram. Of the 164 skull base procedures performed during the study period, 58 pure endoscopic endonasal procedures were excluded. Among the remaining 106 exoscope-assisted procedures, neuroendoscopic assistance was used in 42 cases (39.6%).

This retrospective observational study included consecutive exoscope-assisted skull base procedures performed at Nippon Medical School between March 2024 and June 2026. Pure endoscopic endonasal procedures, including routine pituitary adenoma surgery, were excluded. A total of 164 skull base procedures were performed during the study period. After exclusion of 58 pure endoscopic endonasal procedures, 106 exoscope-assisted skull base procedures were identified. Neuroendoscopic assistance was used in 42 of these 106 procedures (39.6%), each performed in a different patient, and these 42 procedures constituted the neuroendoscopy-assisted cohort. None of the 106 exoscope-assisted procedures required conversion to or combined use of a conventional operating microscope. The study selection process is summarized in Figure 1.

### 2.2. Data Collection

Clinical and operative data, including age, sex, pathology, surgical approach, indication for neuroendoscopic assistance, postoperative neurological outcomes, and complications potentially related to neuroendoscope insertion or manipulation, were retrospectively reviewed from medical records and operative video recordings. Complete operative video recordings were available for all 42 neuroendoscopy-assisted procedures.

### 2.3. Exoscopic and Neuroendoscopic Systems and Operative Setup

All procedures were performed using the ORBEYE three-dimensional exoscope system (Olympus, Tokyo, Japan) as the primary visualization system. A rigid 30° endoscope (A81011A; 2.7 mm outer diameter, 137 mm working length; Olympus, Tokyo, Japan) was selectively introduced under direct visualization when the operating surgeon considered the exoscopic view insufficient for adequate inspection of critical anatomical structures or surgical targets. The decision to introduce the neuroendoscope was made intraoperatively by the operating surgeon. The general criteria for neuroendoscopic assistance remained unchanged throughout the study period.

The neuroendoscope was introduced under direct visualization and stabilized using an EndoArm holding system (Olympus, Tokyo, Japan), allowing visualization of anatomical structures outside the direct exoscopic line of sight while preserving bimanual surgical manipulation. Depending on the surgical requirements, exoscopic and endoscopic images were displayed simultaneously using either picture-in-picture or side-by-side display modes. Other intraoperative adjuncts, including indocyanine green videoangiography, micro-Doppler ultrasonography, and electrophysiological monitoring, were used as clinically indicated. The indications for neuroendoscopic assistance were categorized as follows: (1) clip and perforator confirmation during aneurysm surgery; (2) inspection of the optic canal; (3) visualization of Meckel’s cave; (4) simultaneous transcranial and endonasal surgery; and (5) visualization of lesions located behind cranial nerves. Each procedure was assigned to a single category according to the primary indication for neuroendoscopic assistance. When more than one potential indication was present, the category corresponding to the principal reason for introducing the neuroendoscope was recorded.

### 2.4. Assessment of Neuroendoscopic Contribution

Complete operative video recordings from all 42 neuroendoscopy-assisted procedures were retrospectively reviewed by two neurosurgeons (F.M. and M.T.) to assess the observed intraoperative contribution of neuroendoscopy. Based on the operative findings, the contribution was categorized into three levels: (1) visualization-only, when neuroendoscopy provided additional visualization of anatomical structures not adequately visualized with the exoscope but did not confirm a specific surgical objective or alter the planned maneuver; (2) confirmatory, when neuroendoscopy provided additional confirmation of critical anatomy or the intended surgical result without changing the planned maneuver; and (3) decision-changing, when neuroendoscopic findings directly resulted in modification of the surgical maneuver or strategy. Final classification was determined by consensus between the two reviewers.

### 2.5. Clinical Outcomes and Complications

Postoperative clinical and imaging outcomes were retrospectively assessed according to pathology. In aneurysm procedures, postoperative neurological status and diffusion-weighted magnetic resonance imaging findings were evaluated. MRI, including diffusion-weighted imaging, was performed on postoperative days 1 and 7 in all 30 neuroendoscopy-assisted aneurysm procedures. Modified Rankin Scale scores were used to assess functional outcome.

For tumor procedures, postoperative neurological and cranial nerve outcomes and complications were reviewed. Particular attention was paid to events potentially attributable to insertion or manipulation of the neuroendoscope, including cranial nerve injury, vascular injury, and brain contusion. Complications were considered neuroendoscopy-related when the operative video and clinical course indicated a direct temporal and anatomical relationship to neuroendoscope insertion or manipulation.

### 2.6. Statistical Analysis

Given the descriptive nature of this study and the small, heterogeneous pathology-specific subgroups, no inferential statistical testing was performed. Continuous variables are presented as mean ± standard deviation (range), and categorical variables as numbers and percentages. The analysis was intended to characterize patterns of neuroendoscopic use and its observed intraoperative contribution rather than to assess comparative efficacy between neuroendoscopy-assisted and exoscope-only procedures.

## 3. Results

### 3.1. Patient Characteristics

A total of 164 skull base procedures were performed during the study period. After excluding 58 pure endoscopic endonasal procedures, 106 exoscope-assisted skull base procedures were analyzed, including 57 aneurysms, 24 meningiomas, 14 schwannomas, 5 orbital tumors, 2 chordomas, and 4 other lesions. Neuroendoscopic assistance was used in 42 of the 106 procedures (39.6%), each performed in a different patient. The neuroendoscopy-assisted cohort comprised 27 women (64.3%) and 15 men (35.7%), with a mean age of 67.0 ± 12.5 years (range, 31–85 years). The pathological diagnoses included 30 aneurysms, 6 meningiomas, 4 schwannomas, 1 chordoma, and 1 chordoid glioma. Patient characteristics and pathological diagnoses are summarized in Table 1.

### 3.2. Indications for Neuroendoscopic Assistance

The indications for neuroendoscopic assistance are summarized in Table 2. The most common indication was clip and perforator confirmation during aneurysm surgery (30/42, 71.4%), followed by inspection of the optic canal (4/42, 9.5%), visualization of Meckel’s cave (3/42, 7.1%), simultaneous transcranial and endonasal surgery (3/42, 7.1%), and visualization of lesions located behind cranial nerves (2/42, 4.8%). Neuroendoscopic assistance was used in 30 of 57 aneurysm procedures (52.6%), 6 of 24 meningioma procedures (25.0%), 4 of 14 schwannoma procedures (28.6%), and 1 of 2 chordoma procedures (50.0%). The remaining neuroendoscopy-assisted procedure involved a chordoid glioma.

### 3.3. Observed Contribution of Neuroendoscopic Assistance

Complete operative video recordings were available for all 42 neuroendoscopy-assisted procedures and were retrospectively reviewed. The observed contribution of neuroendoscopy was classified as visualization-only in 8 of 42 procedures (19.0%), confirmatory in 20 (47.6%), and decision-changing in 14 (33.3%). Thus, neuroendoscopy provided either confirmatory or decision-changing information in 34 of 42 procedures (81.0%).

Among the 14 decision-changing procedures, neuroendoscopic findings led to clip repositioning in 5 aneurysm procedures and additional tumor removal in 9 tumor procedures. The observed intraoperative contribution of neuroendoscopic assistance is summarized in Table 3.

### 3.4. Representative Applications

**Figure 2:**
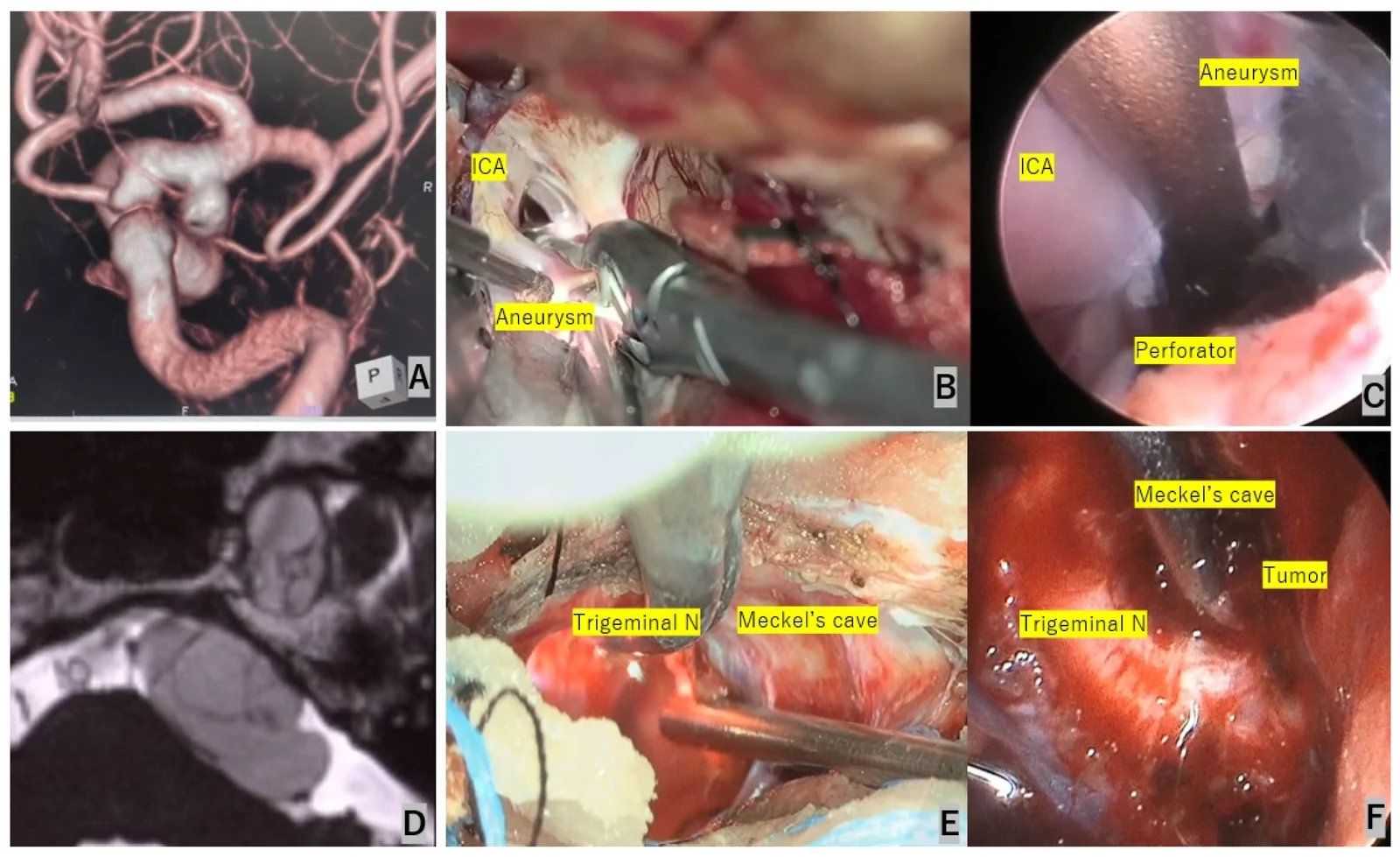
Representative applications of neuroendoscopic assistance during aneurysm and Meckel’s cave surgery. (**A**–**C**) Clip and perforator confirmation during clipping of an intracranial aneurysm. (**A**) Preoperative three-dimensional computed tomography angiography demonstrating the aneurysm. (**B**) Exoscopic view during aneurysm clipping. (**C**) Endoscopic view providing additional visualization of the aneurysm neck and perforating arteries hidden from the direct exoscopic line of sight. (**D**–**F**) Visualization of Meckel’s cave during resection of a trigeminal schwannoma. (**D**) Preoperative T2WI magnetic resonance imaging showing the tumor extending into Meckel’s cave. (**E**) Exoscopic intraoperative view. (**F**) Endoscopic view allowing inspection of Meckel’s cave and visualization of tumor portions hidden from the direct exoscopic line of sight. ICA, internal carotid artery; Trigeminal N, trigeminal nerve.

**Figure 3:**
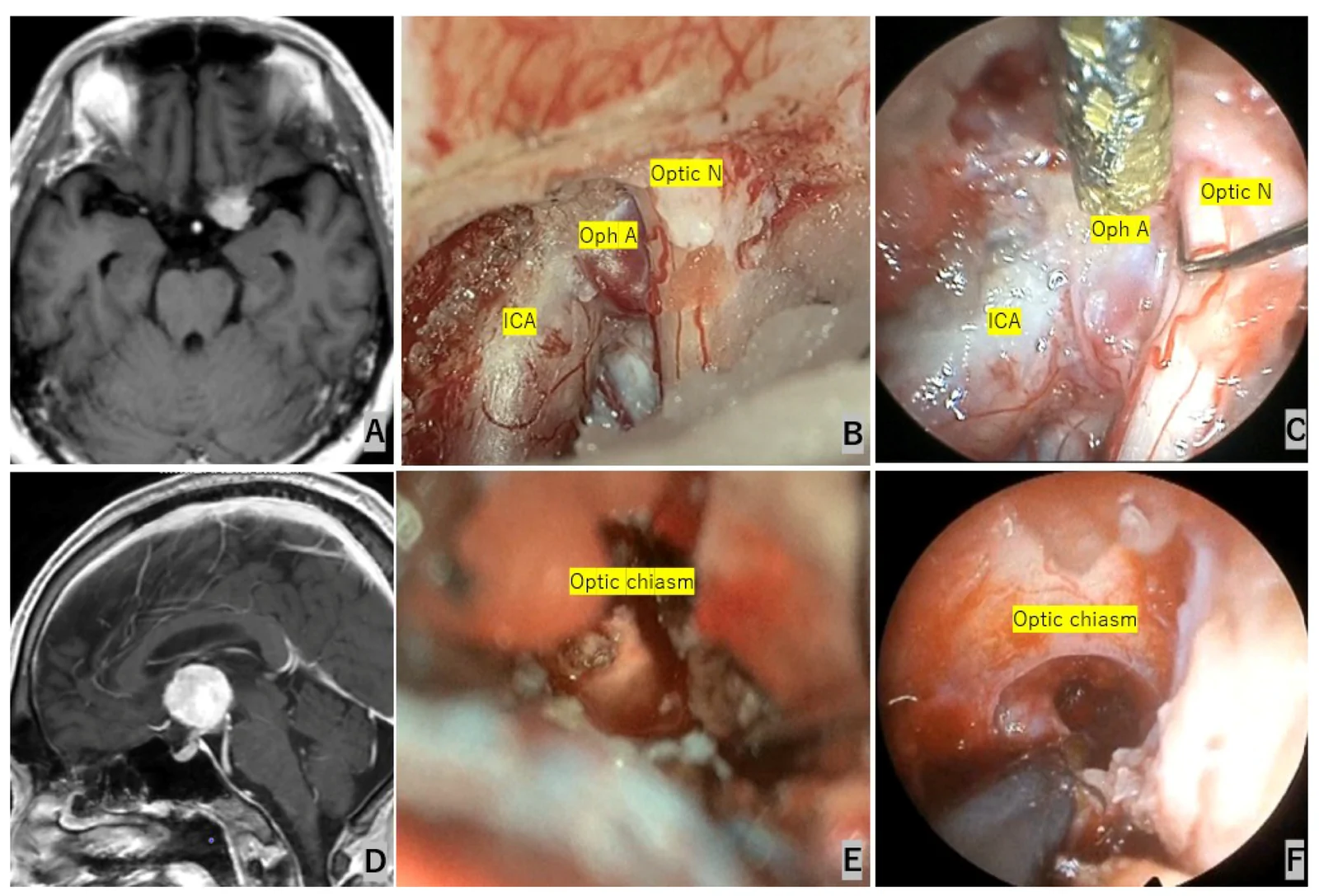
Representative applications of neuroendoscopic assistance during optic canal inspection and simultaneous transcranial-endonasal surgery. (**A**–**C**) Inspection of the optic canal during resection of a medial sphenoid wing meningioma. (**A**) Preoperative contrast-enhanced magnetic resonance imaging demonstrating tumor extension toward the optic canal. (**B**) Exoscopic intraoperative view. (**C**) Endoscopic view providing enhanced visualization of the optic canal and its contents. (**D**–**F**) Simultaneous transcranial and endonasal surgery for a chordoid glioma. (**D**) Preoperative contrast-enhanced magnetic resonance imaging demonstrating a third ventricular chordoid glioma. (**E**) Exoscopic view obtained through the transcranial corridor. (**F**) Endoscopic view obtained through the endonasal corridor, allowing real-time integration of information from both surgical trajectories. Oph A, ophthalmic artery; Optic N, optic nerve.

Representative applications of neuroendoscopic assistance are shown in Figure 2 and Figure 3 and Supplementary Video S1. In aneurysm surgery, angled neuroendoscopic visualization allowed inspection of perforating arteries and clip positioning beyond the direct exoscopic line of sight. In skull base tumor surgery, neuroendoscopy provided additional visualization of anatomically restricted regions, including the optic canal, Meckel’s cave, and areas behind cranial nerves. Neuroendoscopy was also used during simultaneous transcranial and endonasal procedures to provide complementary visualization from different surgical corridors.

### 3.5. Clinical and Postoperative Outcomes

Among the 30 patients who underwent neuroendoscopy-assisted aneurysm surgery, postoperative magnetic resonance imaging, including diffusion-weighted imaging, was performed in all patients on postoperative days 1 and 7. No new diffusion-restricted lesions suggestive of perforator infarction were identified on either examination, and no deterioration in modified Rankin Scale score was observed.

Among the 12 patients who underwent neuroendoscopy-assisted tumor surgery, postoperative neurological and cranial nerve outcomes were reviewed. No complications judged attributable to neuroendoscope insertion or manipulation, including cranial nerve injury, vascular injury, or brain contusion, were identified. Overall, no complications judged attributable to neuroendoscope insertion or manipulation were identified among the 42 neuroendoscopy-assisted procedures (0/42; two-sided 95% exact binomial CI, 0–8.4%). No such complications were identified in either the aneurysm subgroup (0/30; 95% CI, 0–11.6%) or the tumor subgroup (0/12; 95% CI, 0–26.5%).

## 4. Discussion

In this retrospective single-center series, neuroendoscopic assistance was used in 42 of 106 exoscope-assisted skull base procedures (39.6%). The most common indication was confirmation of clip positioning and perforating arteries during aneurysm surgery, followed by visualization of anatomically restricted regions such as the optic canal, Meckel’s cave, and areas behind cranial nerves. Retrospective review of complete operative video recordings demonstrated different levels of intraoperative contribution: visualization-only in 8 procedures (19.0%), confirmatory in 20 (47.6%), and decision-changing in 14 (33.3%).

Importantly, the contribution of neuroendoscopy extended beyond additional visualization in a subset of procedures. Neuroendoscopic findings directly altered the surgical maneuver in 14 procedures, resulting in clip repositioning in 5 aneurysm procedures and additional tumor removal in 9 tumor procedures. These observations provide a more concrete measure of the intraoperative contribution of neuroendoscopy than visualization alone. However, because neuroendoscopy was selectively introduced according to intraoperative anatomical requirements and surgeon judgment, these findings should not be interpreted as evidence that neuroendoscopic assistance improves aneurysm occlusion rates or extent of tumor resection compared with exoscopy alone.

**Figure 4:**
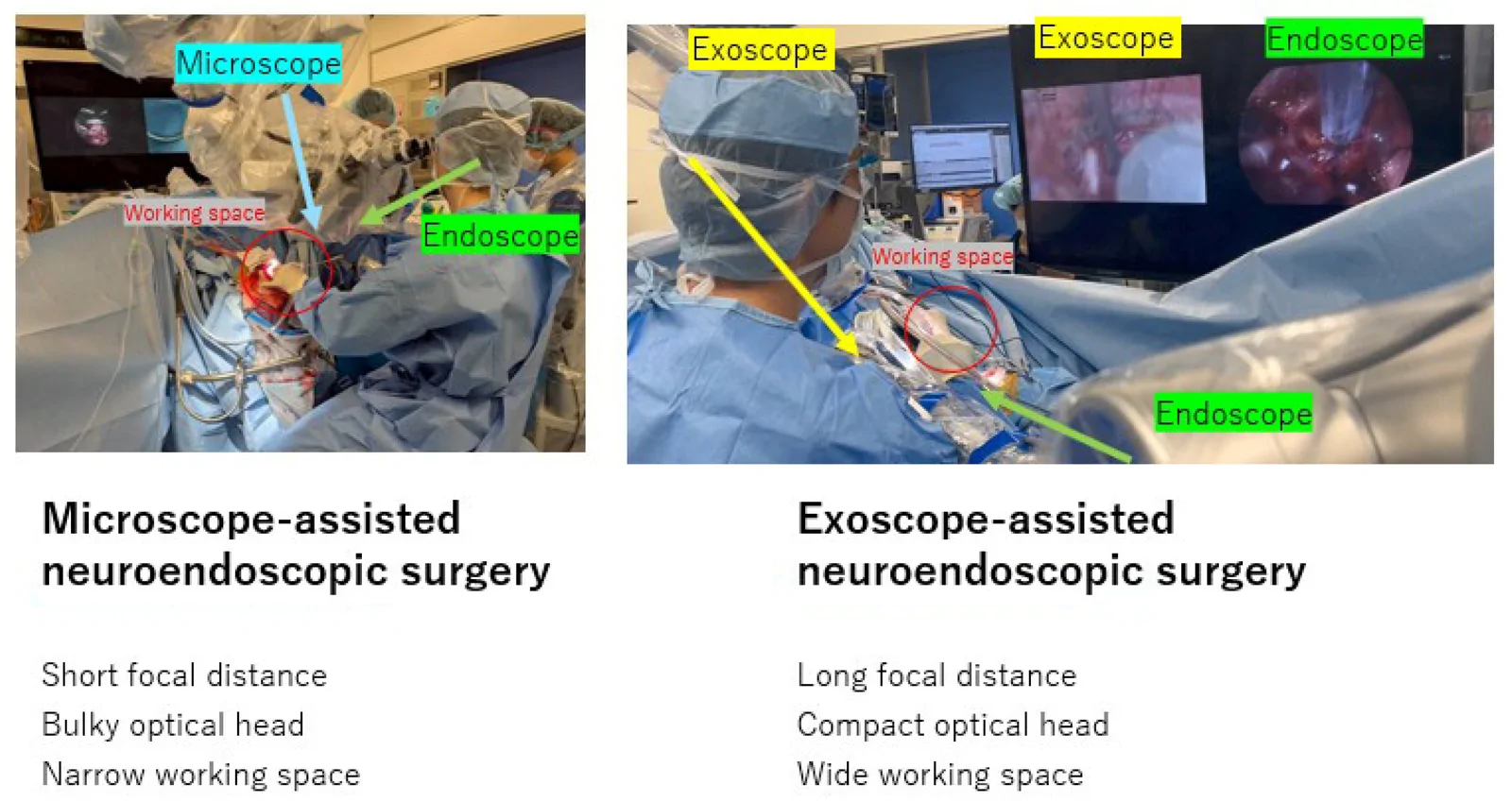
Operative setup for combined exoscopic and neuroendoscopic visualization. The exoscope provides working space for introduction and manipulation of the neuroendoscope, while exoscopic and endoscopic images can be displayed simultaneously on external monitors. The figure illustrates the spatial arrangement of the visualization systems and operative field used in our practice.

The present findings suggest that the advantages of neuroendoscopy may be further enhanced in the exoscope era. Compared with conventional operating microscopes, exoscopes provide a longer working distance and a smaller optical head, resulting in a wider working space for the introduction and manipulation of neuroendoscopes [5,8,9]. Furthermore, modern exoscopic systems allow simultaneous display of exoscopic and endoscopic images using either picture-in-picture or side-by-side display modes, permitting concurrent visualization of both image sources (Figure 4) [4,6]. These technical characteristics may facilitate integration of neuroendoscopy into exoscope-assisted skull base surgery.

The value of angled endoscopic visualization in neurosurgery has been reported in both aneurysm and skull base surgery. In aneurysm surgery, endoscopic assistance can provide additional visualization of perforating arteries, the parent artery, and structures located behind the aneurysm or clip blades that may remain outside the direct microscopic line of sight [10]. Similarly, in skull base tumor surgery, endoscopic assistance has been used to inspect anatomically concealed regions and identify residual tumor beyond the direct operative view [11,12]. Linsler et al. [13] evaluated endonasal endoscopic and endoscope-assisted transcranial approaches for Rathke’s cleft cysts, emphasizing the potential relationship between surgical visualization, extent of resection, and recurrence. Kanczok et al. [14] also reported that endoscopic assistance during microsurgical skull base meningioma resection identified residual tumor not apparent under microscopic visualization, particularly in posterior fossa lesions. These findings support the concept that angled endoscopic visualization may provide complementary anatomical information in surgically restricted corridors.

The present study differs from previous reports primarily focused on endoscope-assisted microscopic surgery by examining neuroendoscopic assistance specifically within an exoscope-based operative environment. The exoscope and neuroendoscope share an external monitor-based visualization platform, allowing the two image sources to be displayed simultaneously using picture-in-picture or side-by-side configurations. In our experience, this configuration facilitated transition between exoscopic and endoscopic views without requiring a change in the primary visualization platform. Nevertheless, operative time, workflow interruption, surgeon ergonomics, and usability were not quantitatively assessed in the present study; therefore, potential workflow or ergonomic advantages of this configuration remain hypotheses requiring prospective evaluation.

Previous reports of combined exoscopic and endoscopic surgery have demonstrated the technical feasibility of this approach. Murai et al. [6] described preliminary experience with temporary simultaneous endoscopic use during exoscopic neurosurgery, whereas Yano et al. [7] reported endoscope-assisted skull base surgery under exoscopic visualization. Importantly, no patients in the present cohort overlapped with those reported by Murai et al. The present study extends these earlier technical observations by evaluating a consecutive exoscope-assisted skull base cohort and by characterizing the indications and observed intraoperative contribution of neuroendoscopic assistance.

**Figure 5:**
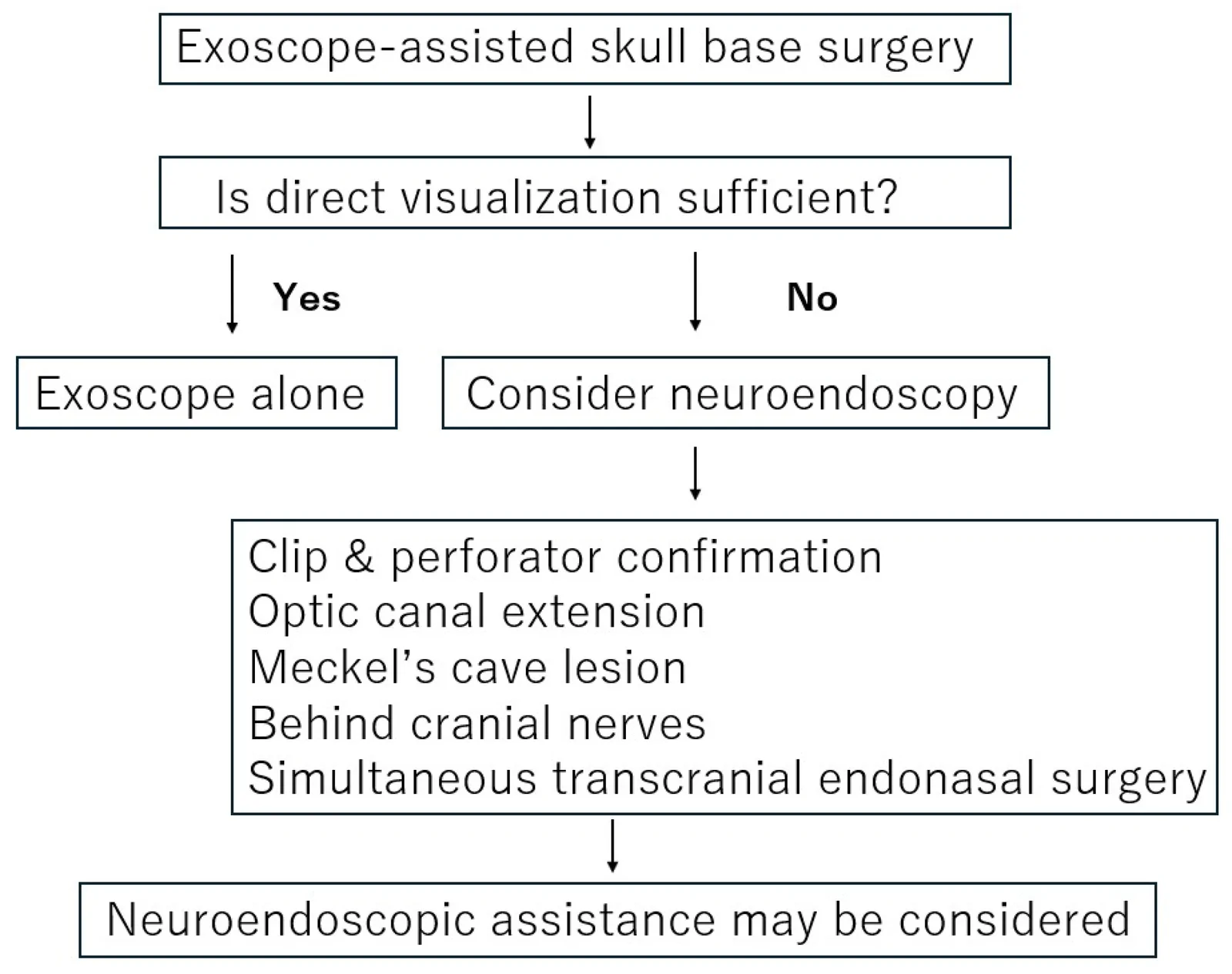
Experience-derived decision framework for neuroendoscopic assistance during exoscope-assisted skull base surgery. Based on the findings of the present study, neuroendoscopic assistance may be considered when anatomical structures are hidden from the direct exoscopic line of sight. Representative indications include clip and perforator confirmation during aneurysm surgery, inspection of the optic canal, visualization of Meckel’s cave, simultaneous transcranial and endonasal surgery, and visualization of lesions located behind cranial nerves. This framework was derived from our institutional experience and has not been externally validated.

Based on the findings of the present study, neuroendoscopic assistance may be particularly useful in situations in which anatomical structures are hidden from the direct exoscopic line of sight. We therefore propose an experience-derived decision framework (Figure 5) illustrating clinical situations in which neuroendoscopic assistance may be considered. In aneurysm surgery, neuroendoscopy facilitates confirmation of perforating arteries and clip placement [15,16,17]. In skull base surgery, it enables inspection of the optic canal, visualization of Meckel’s cave, and assessment of lesions extending behind cranial nerves. In addition, neuroendoscopy may provide complementary visualization during simultaneous transcranial and endonasal surgery by allowing information obtained from both surgical corridors to be integrated in real time. This framework is derived from our institutional experience and should be regarded as descriptive and hypothesis-generating rather than as a validated recommendation.

Despite its advantages, neuroendoscopic assistance requires careful handling to avoid neurovascular injury. Blind insertion should be avoided, and the endoscope should be introduced under direct visualization whenever possible. In the present series, all endoscopes were stabilized using an EndoArm holding system, allowing stable endoscopic visualization while preserving bimanual operative manipulation [3]. Importantly, all patients undergoing neuroendoscopy-assisted aneurysm surgery underwent postoperative magnetic resonance imaging, including diffusion-weighted imaging, on postoperative day 1 and postoperative day 7. No new ischemic lesions suggestive of perforator infarction or deterioration in functional outcome were observed. In addition, no complications judged attributable to neuroendoscope insertion or manipulation, including cranial nerve injury, vascular injury, or brain contusion, were identified in tumor surgery. Overall, no complications judged attributable to neuroendoscope insertion or manipulation were identified in this series; however, the limited sample size precludes definitive conclusions regarding the safety of neuroendoscopic assistance. Future multicenter studies are warranted to validate these findings and further refine the indications for neuroendoscopic assistance during exoscope-assisted skull base surgery.

## 5. Limitations

This study has several limitations. First, it was a retrospective, single-center study with a relatively small cohort. In particular, the pathology-specific subgroups were small, especially among tumor cases, precluding meaningful subgroup-specific inferential analyses of extent of resection, recurrence, neurological outcomes, or other clinical endpoints. Second, neuroendoscopic assistance was introduced selectively according to intraoperative anatomical requirements and surgeon judgment; therefore, the 64 exoscope-only procedures did not constitute a prespecified comparator group, and direct comparison between the two groups would be susceptible to confounding by indication. Third, although complete operative video recordings allowed retrospective classification of neuroendoscopic contribution, this classification was not prospectively defined or independently validated. Fourth, operative time, workflow interruption, ergonomics, and usability were not quantitatively assessed. Finally, the proposed decision framework is derived from our institutional experience and has not been externally validated. The present findings should therefore be interpreted as descriptive and hypothesis-generating rather than as evidence of comparative efficacy.

## 6. Conclusions

Neuroendoscopic assistance provided additional intraoperative information during exoscope-assisted skull base surgery, with decision-changing findings identified in 14 of 42 procedures. No complications judged attributable to neuroendoscope insertion or manipulation were identified in this series; however, the limited sample size precludes definitive conclusions regarding safety. These findings support further investigation of selective neuroendoscopic assistance for anatomical regions that are difficult to visualize directly with the exoscope.

## Figures and Tables

**Table 1:** Patient characteristics and pathological diagnoses of the study cohort.

Variable	Value
Number of patients	42
Age, years, mean ± SD (range)	67.0 ± 12.5 (31–85)
Female sex, *n* (%)	27 (64.3)
Pathological diagnosis, *n* (%)	
Intracranial aneurysm	30 (71.4)
Meningioma	6 (14.3)
Schwannoma	4 (9.5)
Chordoma	1 (2.4)
Chordoid glioma	1 (2.4)

**Table 2:** Indications for neuroendoscopic assistance during exoscope-assisted skull base surgery.

Indication	*n* (%)
Clip and perforator confirmation during aneurysm surgery	30 (71.4)
Inspection of the optic canal	4 (9.5)
Visualization of Meckel’s cave	3 (7.1)
Simultaneous transcranial and endonasal surgery	3 (7.1)
Visualization of lesions located behind cranial nerves	2 (4.8)
Total	42 (100)

**Table 3:** Observed intraoperative contribution of neuroendoscopic assistance.

Contribution Category	*n* (%)	Definition or Observed Consequence
Visualization-only	8 (19.0)	Additional visualization without confirmation or change in surgical maneuver
Confirmatory	20 (47.6)	Confirmation of critical anatomy or intended surgical result without change in surgical maneuver
Decision-changing	14 (33.3)	Modification of the surgical maneuver based on neuroendoscopic findings
Clip repositioning	5 (11.9)	Clip repositioning during aneurysm surgery
Additional tumor removal	9 (21.4)	Additional tumor removal after neuroendoscopic inspection
Total	42 (100)	

## Data Availability

The data presented in this study are available from the corresponding author upon reasonable request. The data are not publicly available due to institutional privacy regulations and the protection of patient confidentiality.

## Supplementary Materials

The following supporting information can be downloaded at https://www.mdpi.com/article/10.3390/brainsci16090942/s1. Video S1: Representative applications of neuroendoscopic assistance during exoscope-assisted skull base surgery. The video demonstrates four representative indications for neuroendoscopy: (A) clip and perforator confirmation during aneurysm surgery, (B) visualization of Meckel’s cave, (C) inspection of the optic canal, and (D) simultaneous transcranial and endonasal surgery.

## Abbreviations

PIP, picture-in-picture; ORBEYE, Olympus Orbeye three-dimensional exoscope system; EEA, endoscopic endonasal approach.
